# An oversimplification of physiological principles leads to flawed macroecological analyses

**DOI:** 10.1002/ece3.5721

**Published:** 2019-10-21

**Authors:** Justin G. Boyles, Danielle L. Levesque, Julia Nowack, Michał S. Wojciechowski, Clare Stawski, Andrea Fuller, Ben Smit, Glenn J. Tattersall

**Affiliations:** ^1^ Cooperative Wildlife Research Laboratory Center for Ecology School of Biological Sciences Southern Illinois University Carbondale IL USA; ^2^ School of Biology and Ecology University of Maine Orono ME USA; ^3^ School of Biological and Environmental Sciences Liverpool John Moores University Liverpool UK; ^4^ Department of Vertebrate Zoology and Ecology Faculty of Biology and Environmental Protection Nicolaus Copernicus University Toruń Poland; ^5^ Department of Biology Norwegian University of Science and Technology Trondheim Norway; ^6^ Brain Function Research Group School of Physiology University of the Witwatersrand Johannesburg South Africa; ^7^ Department of Zoology and Entomology Rhodes University Grahamstown South Africa; ^8^ Department of Biological Sciences Brock University St. Catharines ON Canada

## Abstract

Macrophysiological analyses are useful to predict current and future range limits and improve our understanding of endotherm macroecology, but such analyses too often rely on oversimplifications of endothermic thermoregulatory and energetic physiology, which lessens their applicability. We detail some of the major issues with macrophysiological analyses based on the classic Scholander–Irving model of endotherm energetics in the hope that it will encourage other research teams to more appropriately integrate physiology into macroecological analyses.

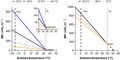

In light of the rapidly changing climate, there is an urgent need to develop a mechanistic understanding of how physiological functioning mediates ecological patterns. Recently, there has been a spate of papers using analyses that scale up from a standard physiological model, the Scholander–Irving model, to make predictions about range constraints on endothermic vertebrates (Buckley, Khaliq, Swanson, & Hof, 2018; Fristoe et al., 2015; Khaliq, Böhning‐Gaese, Prinzinger, Pfenninger, & Hof, 2017; Khaliq, Hof, Prinzinger, Böhning‐Gaese, & Pfenninger, 2014). Here, we argue that oversimplifications of the Scholander–Irving model and the use of questionable datasets lead to questionable macrophysiological analyses. Many of these problems have been addressed elsewhere, directly and indirectly (e.g., McKechnie, Coe, Gerson, & Wolf, 2017; Mitchell et al., 2018), although the focus has largely been on the applicability of the Scholander–Irving model to warm environmental temperatures, which are often seen as more relevant to climate change. However, one specific aspect of the Scholander–Irving model, the assumption that energy expenditure of an endotherm below the thermoneutral zone (TNZ) can be described by basic Newtonian physics, has been used incorrectly in several papers. While not the only paper based on this assumption, the recent work by Buckley et al. (2018) reinvigorated discussions among physiologists about improper interpretations of the Scholander–Irving model. Our concerns are not new and have been voiced repeatedly in the past (Calder & Schmidt‐Nielsen, 1967; King, 1964; Tracy, 1972), but many of these ideas seem to have been buried by time. Our goal here is to bring these concerns back to the forefront in the context of modern large‐scale macrophysiological analyses, using the work of Buckley et al. (2018) as an example where relevant. We detail these ideas below, but King (1964) provided a scathing, yet technically accurate summary of our position over five decades ago: “The convenience of the Newtonian model as a heuristic or pedagogical device is readily apparent; but its use as an analytical instrument to reveal relatively small interspecific or seasonal adaptations in metabolism is a practice which is beset by many uncertainties, and which occasionally appears to encourage a Procrustean fitting of data.”

In practice, endothermic vertebrates pose significant challenges in large‐scale ecological analyses because the relationship between environmental conditions and functional energetics is mediated by complex metabolic and thermoregulatory control (Levesque, Nowack, & Stawski, 2016). At the heart of the issue is the reliance on the Scholander–Irving model (Scholander, Hock, Walters, Johnson, & Irving, 1950), a classic descriptive model of the relationship between ambient temperature and metabolic rate *in strict homeotherms* (i.e., species that maintain their body temperature within a somewhat narrow, although undefined range). Under this model, homeothermic endotherms are assumed to maintain a constant basal metabolic rate within the TNZ, a constant body temperature (*T*_b_), and a constant thermal conductance. At ambient temperatures below the lower critical temperature (*T*_lc_, the lower boundary of the TNZ), metabolic rate increases to compensate for increased heat loss and to maintain constant *T*_b_. Importantly, these relationships vary with many factors, including season, so values measured during summer are inappropriate for analyses of cold tolerance during winter. While the Scholander–Irving model is important for descriptive analyses of energetic function in homeothermic endotherms, its direct application to modeling environmental temperature thresholds for most endotherms is questionable (Levesque et al., 2016; Mitchell et al., 2018; Porter & Kearney, 2009). To generalize across large geographic scales, many analyses of endothermic energetics rely on a series of simplifying—and often unjustified—assumptions (reviewed by Mitchell et al., 2018). These simplifying assumptions are common, but an overreliance on them has inhibited a mechanistic understanding of global patterns in endothermic physiology. Relevant to the current discussion, the Scholander–Irving model predicts that a regression describing the relationship between metabolic rate and ambient temperatures below the *T*_lc_ extrapolates to an ambient temperature equal to *T*_b_, if metabolic heat production was to reach zero (Scholander et al., 1950). This idea essentially requires that heat balance in endotherms follows Newton's laws of cooling, which may be a reasonable simplification in a small number of homeothermic species (usually mammals, but not birds), but is clearly not universal (King, 1964; McNab, 1979). There are numerous problems, both biological and computational, with this approach. First, a line fit through metabolic rate data rarely predicts *T*_b_ accurately, often overestimating it by as much as 10°C (Calder & Schmidt‐Nielsen, 1967; McNab, 1979). As a simple example, we calculated *T*_b_ using the relationship between metabolic rate and *T*_lc_ for the rock pocket mouse (*Chaetodipus intermedius*), one of the species included in Buckley et al. (2018). In the source paper (Bradley, Yousef, & Scott, 1975), *T*_b_ is estimated at ~35°C and metabolic data are provided for two periods: January and April. By extrapolating the metabolic rate line to zero on the *y*‐axis, *T*_b_ is estimated at 37.9°C in January and 45.7°C in April (Figure 1). Clearly, the April value is unrealistic for a mammal. The inverse assumption that *T*_b_ can be used to predict metabolic rate is also problematic. For example, a difference in estimate of only 1°C (the black line vs. the gray line on the left panel of Figure 1) for the *T*_b_ of the four‐toed hedgehog (*Atelerix albiventris*) would lead to a difference in the metabolic rate at the cold range boundary (MR_CRB_) described by Buckley et al. (2018) of ~12%.

**Figure 1:**
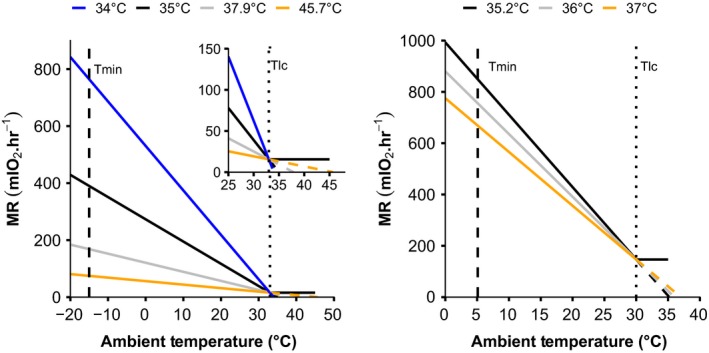
Demonstration of the inherent limitations of using body temperature (*T*_b_) and the lower critical temperature (*T*_lc_) of the thermal neutral zone to calculate thermal conductance (C) when *T*_b_ and *T*_lc_ are poorly defined. Buckley et al. (2018) analyzed metabolic expansibility, which is the metabolic rate at the range boundary (MR_RB_) divided by basal metabolic rate (BMR). MR_RB_, and therefore ME, changes drastically depending on assumptions of *T*_b_ and *T*_lc_ used to calculate C. Data are for a rock mouse (*Chaetodipus intermedius*, left; Bradley et al., 1975) and a hedgehog (*Atelerix albiventris*, right; McNab, 1980). In each case, black lines represent the *T*_b_ value used by Buckley et al and the various other lines represent plausible assumptions about *T*_b_, assuming *T*_lc_ is constant. Dashed lines represent the extrapolation of *T*_b_ from the MR line assuming the animal is perfectly following Newton's Laws of Cooling. The inset in (a) is an expanded view encapsulating the *T*_lc_ and *T*_b_ of the rock mouse

The Scholander–Irving model was groundbreaking in 1950 and has proven to be useful in shaping our basic understanding of endothermic thermoregulation (Somero, 2013). However, this model requires that (a) thermal conductance below the TNZ is constant, (b) that endotherms maintain a relatively constant *T*_b_, and (c) that *T*_lc_ can be estimated at a single value. The idea of a constant thermal conductance has long been discounted (Tracy, 1972) and has repeatedly been shown to be unrealistic (e.g., Calder & Schmidt‐Nielsen, 1967; Noakes, Smit, Wolf, & McKechnie, 2013). Large‐scale analyses assuming Newtonian cooling are on shaky theoretical footing if the assumption of constant thermal conductance is violated (King, 1964). Scholander et al. (1950) also assume that most mammals maintain *T*_b_ within ±1°C. While the data are not strictly analogous, such homeothermy likely occurs in <30% of mammals with quality *T*_b_ data collected during winter (Boyles et al., 2013). Even strictly homeothermic humans show a decrease of ~1°C during sleep (Wright, Badia, Myers, Plenzler, & Hakel, 1997) and *T*_b_ of marsupials and monotremes can be lowered by >8°C during normothermic resting. Further, describing endothermic *T*_b_ is surprisingly difficult, and the most commonly used metric, mean *T*_b_, is usually a poor descriptor of regulated endothermic *T*_b_ (Boyles, 2019; Hetem, Maloney, Fuller, & Mitchell, 2016). Finally, *T*_lc_ is often difficult to delineate at a single temperature using standard respirometry techniques, because no clear “breakpoint” in metabolic rate exists for many species (McNab, 1995), and *T*_lc_ may change seasonally (Kobbe, Nowack, & Dausmann, 2014). Even ignoring these theoretical concerns, extreme care must be taken in extrapolating from such variable and hard‐to‐describe values.

If one begins with the assumption the Scholander–Irving model is static and easily fit for all endotherms, data quality issues are nearly inevitable. Here, we use the dataset of Buckley et al. (2018) as an example to demonstrate how poor assumptions can be manifested in poor data quality. Specifically, we traced *T*_b_ and *T*_lc_ data for a subset of their dataset back to the original sources. As with previous critiques of the upper critical temperature in similar datasets (McKechnie et al., 2017), we found considerable variability in the quality of the data used (Table 1). For example, many of the values presented were simply one value within the range of *T*_b_ or *T*_lc_ listed in the paper, but there was little consistency in how the value was chosen. This error stems from the assumption that endotherms maintain single, constant *T*_b_s. Likewise, many older papers calculated *T*_lc_ by eyeballing lines through metabolic data and estimating the intersection with basal metabolic rate. Therefore, the values are approximations (in many cases, they are different than what we would estimate from the same data). This error stems from the assumption of a single, unchanging *T*_lc_. Two of us (JGB and DLL) independently judged the quality of the *T*_b_ and *T*_lc_ included in the first 20 mammal species included by Buckley et al. (2018) (excluding species with partial data and including only one species from a genus; Table 1). Of those 20 datasets, we classified five as appropriate for inclusion in the analysis (i.e., data generally followed the Scholander–Irving model, and *T*_b_ variation was relatively low). Note that even among these “appropriate” datasets, data for three of these five species were collected on zoo animals and may therefore not represent wild animals. We classified nine as marginal or questionable for inclusion in the dataset (i.e., *T*_b_ varied by 3°C or *T*_lc_ was difficult to determine). Ultimately, we classified six as inappropriate for inclusion (i.e., there were clear violations of the Scholander–Irving model or the citation was incorrect and impossible to track). Importantly, authors of several of the original papers commented that either *T*_b_ or *T*_lc_ was difficult or impossible to establish for several of these species (e.g., *Arctictis binturong*, McNab, 1995). Such datasets are too often compiled by ignoring biologically important variation and shoehorning incompatible data into a highly conceptualized model (King, 1964).

**Table 1:** Evaluation of data quality of the first 20 mammal species included in the analysis of Buckley et al. (2018)

Species	*T*_b_	*T*_lc_	Original source	Comments	*n*	Wild/Zoo/Lab	Data quality
*Abrothrix andinus*	39.3	26.8	Bozinovic and Rosenmann (1988)	*T*_b_ calculated working backwards estimates of MR. Actual measured *T*_b_ differs (and has a range)	8	Wild	Questionable
*Ailurus fulgens*	37.6	25	McNab (1988)	McNab argues that they do not follow standard homeotherm energetics; Scholander‐Irving (S‐I) model likely a bad model for this species	2	Zoo	Questionable
*Akodon azarae*	37.7	30	Dalby and Heath (1976)	*T*_b_ continues to drop with *T*_a_, suggesting the S‐I model might be inappropriate	8	Lab	Questionable
*Ammospermophilus leucurus*	37.2	31	Dawson (1955)	*T*_b_ used was measured at *T*_a_ below *T*_lc_ presented; Range was 35.6–38.1°C, and taken during active period instead of the inactive period; animals were excitable and did not cope well with captivity	12	Wild	Questionable
*Anoura caudifer*	36.3	26	McNab (1969)	*T*_b_ in fig. 10 range from roughly 33 to 42°C with c.v. of 4.0% around the mean. *T*_lc_ was not referenced in the original paper, probably estimated from fig. 10.	Unknown	Wild	Questionable
*Aplodontia rufa*	38	26.5	McNab (1979)	Appears to follow the S‐I model. There is some minor variation in *T*_b_	3	Wild	Good
*Apodemus mystacinus*	38.3	28	Haim, Rubal, and Harari (1993)	Appears to follow the S‐I model. Relatively low variation in *T*_b_	6	Wild	Good
*Arctictis binturong*	36	27	McNab (1995)	McNab argues the core temperature is variable (~32–41°C) and decreases with ambient temperature ; McNab also says “the zone of thermoneutrality…was especially difficult to define”	2	Zoo	Questionable
*Arctogalidia trivirgata*	36.2	19	McNab (1995)	The S‐I model is inappropriate for this species and *T*_lc_ is impossible to define with the presented data. McNab says “the variability in rate of metabolism made the zone of thermoneutrality difficult to define with clarity”	4	Zoo	Poor
*Artibeus concolor*	35	29	McNab (1969)	Paper lists *T*_b_ of 35.3°C with a c.v. of 4.1%. No reference to *T*_lc_, so probably estimated from fig. 17, and we would estimate it lower (probably about 28°C).	?	Wild	Questionable
*Atelerix albiventris*	35.2	30	McNab (1980)	S‐I model is probably appropriate, given the data presented herein	2	Zoo	Good
*Baiomys taylori*	36	29	Hudson (1965)	Original paper gives a wide range for *T*_b_ (32–36°C) and mentions *T*_lc_ at 29°C in the summary and 30–33°C in the text (but does not define how it was measured)	10	Lab	Poor
*Blarina brevicauda*	38	25	Neal and Lustick (1973)	Thermoneutral zone analyzed as the range within 95% confidence intervals, but the authors say metabolic rate increases below 30°C. *T*_b_ measured in separate experiments	12	Wild	Questionable
*Cabassous centralis*	33.6	27.5	McNab (1980)	S‐I model is probably appropriate, given the data presented herein	3	Zoo	Good
*Caluromys derbianus*	34	26.36	McNab (1978)	Data were presented for 1 wild animal and 3 larger animals from a zoo. *T*_b_ is from the zoo animals (wild individual was 36°C). *T*_lc_ is given as a value somewhere between the zoo and wild individuals and is not mentioned in the paper	4	Wild/Zoo	Poor
*Canis latrans*	36	22	Golightly and Ohmart (1983)	*T*_b_ is difficult to determine in the original paper. Data come from a desert population. The original authors mention that animals near the northern edge of the range (which is far more relevant in the current study) have *T*_lc_ of −10°C (Shield, 2009)	3	Wild	Poor
*Cannomys badius*	36	26.74	McNab (1979)	Original paper lists *T*_lc_ at 27°C. Otherwise, S‐I model is probably appropriate, given the data presented herein	5	Zoo	Good
*Carollia perspicillata*	36.6	29	McNab (1969)	Paper lists *T*_b_ of 36.4°C with a c.v. of 2.8%. *T*_lc_ probably estimated from fig. 11 and we would estimate it lower (probably about 28°C from fig. 11)	Unknown	Wild	Questionable
*Cercartetus nanus*	34.9	31	Unknown	The citation given is incorrect, and we were unable to track the values back to a paper on this species	Unknown	Unknown	Poor
*Cercopithecus mitis*	37	5	Müller, Kamau, and Maloiy (1983)	*T*_b_ given as 36–38.5°C in the original paper, but otherwise unclear where the presented value comes from; one individual maintained *T*_b_ of around 37°C during the active period; 5°C is given as the approximate *T*_lc_. 5°C was the lowest *T*_a_ measured, so it is impossible to determine *T*_lc_	2	Zoo	Poor

Finally, even if one accepts the assumptions of constant *T*_b_, thermal conductance, and *T*_lc_, there are analytical concerns with using these values to make predictions of organismal responses to conditions far beyond the measured values. For example, one could use these values to estimate energetic expenditure at temperatures below the range empirically measured or to estimate range boundaries (Buckley et al., 2018; Root, 1988). However, such extrapolations are highly sensitive to data quality because they often extend far beyond the range of empirically measured data. Such extrapolations mean that small errors in estimating *T*_b_ or *T*_lc_ (which we reiterate are very hard to estimate and rarely a single value) can lead to large errors in the estimated metabolic rates at cold temperatures. Again, we return to the dataset of Buckley et al. (2018) to demonstrate the scope of the possible error in extrapolating far beyond the known data. In some species, the metabolic rate at the range boundary (MR_RB_) may vary by an order of magnitude depending on the chosen *T*_b_ value (Figure 1). At the extreme, a 1°C change in the *T*_b_ value chosen for these extrapolations can lead to differences of over 400% in calculated metabolic expansibility values for some species (although the median difference for all mammals included in the Buckley et al. dataset is 10%). Unfortunately, it is difficult to predict how these errors will be manifested in the interpretations of data. On one hand, we might predict that errors will be largest in high latitude species because the minimum environmental temperature is far below *T*_lc_ (i.e., the extrapolation is more extreme). On the other hand, the calculated thermal conductance value varies strongly when *T*_lc_ and *T*_b_ are close together, which is most likely to occur in tropical and subtropical species. The results of these analyses often seem to corroborate previous analyses using similar methodologies, but as King (1964) and others have recognized, oversimplifying assumptions of the Scholander–Irving model might result in overly simplified and generic results.

Although we strongly encourage the continued integration of physiological ecology and macroecology, we contend that oversimplification of physiological principles can lead to unreliable analyses. In addition, data quality is of utmost important in these analyses, especially in cases where analyses are highly sensitive to variance in input variables. Understanding the thermophysiology of endotherms relies on recognizing a number of caveats which have unfortunately not been widely adopted by nonspecialists (Mitchell et al., 2018). While we recognize the importance of broader macroscale analyses, such studies would benefit from closer collaborations between macroecologists and physiological ecologists as each could help the other better understand the hidden nuances in their respective analyses and move toward a more comprehensive understanding of global patterns.

## CONFLICT OF INTEREST

None declared.

## AUTHOR CONTRIBUTIONS

JGB and DLL conducted the literature review and analyses. All authors contributed to writing the manuscript.

## Data Availability

No original data were collected for this paper. Data used in the analysis can be found associated with Buckley et al. (2018).
